# Comprehensive transcriptomic analysis unveils the interplay of mRNA and LncRNA expression in shaping collagen organization and skin development in Dezhou donkeys

**DOI:** 10.3389/fgene.2024.1335591

**Published:** 2024-02-09

**Authors:** Xinrui Wang, Yongdong Peng, Huili Liang, Muhammad Zahoor Khan, Wei Ren, Bingjian Huang, Yinghui Chen, Shishuai Xing, Yandong Zhan, Changfa Wang

**Affiliations:** Liaocheng Research Institute of Donkey High-Efficiency Breeding, Liaocheng University, Liaocheng, China

**Keywords:** Dezhou donkey, hide gelatin, collagen, RNA-seq, lncRNA, mRNA, KEGG, genetic markers

## Abstract

The primary focus of donkey hide gelatin processing lies in the dermal layer of donkey hide due to its abundant collagen content. However, the molecular mechanism involved in collagen organization and skin development in donkey skin tissue across various developmental stages remains incomplete. The current study aims to investigate the transcriptomic screening of lncRNAs and mRNA associated with skin development and collagen organization across different ages in Dezhou donkeys’ skin. In the pursuit of this objective, we used nine skin tissue samples obtained from Dezhou donkeys at various ages including 8-month fetal stage, followed by 2 and 8 years. RNA-seq analysis was performed for the transcriptomic profiling of differentially expressed genes (DEGs) and lncRNAs associated with skin development in different age groups. Our investigation revealed the presence of 6,582, 6,455, and 405 differentially expressed genes and 654, 789, and 29 differentially expressed LncRNAs within the skin tissues of Dezhou donkeys when comparing young donkeys (YD) vs. middle-aged donkeys (MD), YD vs. old donkeys (OD), and MD vs. OD, respectively. Furthermore, we identified *Collagen Type I Alpha 1 Chain* (*COL1A1*), *Collagen Type III Alpha 1 Chain* (*COL3A1*), and *Collagen Type VI Alpha 5 Chain* (*COL6A5*) as key genes involved in collagen synthesis, with *COL1A1* being subject to cis-regulation by several differentially expressed LncRNAs, including ENSEAST00005041187, ENSEAST00005038497, and MSTRG.17248.1, among others. Interestingly, collagen organizational and skin development linked pathways including Protein digestion and absorption, metabolic pathways, Phosphatidylinositol 3-Kinase-Protein Kinase B signaling pathway (PI3K-Akt signaling pathway), Extracellular Matrix-Receptor Interaction (ECM-receptor interaction), and Relaxin signaling were also reported across different age groups in Dezhou donkey skin. These findings enhance our comprehension of the molecular mechanisms underlying Dezhou donkey skin development and collagen biosynthesis and organization, thus furnishing a solid theoretical foundation for future research endeavors in this domain.

## 1 Introduction

In the context of modern agriculture and the evolving role of donkeys in agricultural production, this discourse delves into the increasing utilization of donkey products, with particular emphasis on the medicinal value attributed to ejiao, derived from donkey hide (Maigari et al., 2020; Goodrum et al., 2022; Yan et al., 2023). It is of particular note that the dermal layer of donkey hide, enriched with collagen, assumes a pivotal role in the preparation of ejiao. The popularity of ejiao in China and among practitioners of traditional Chinese medicine has led to a surge in demand, subsequently causing a substantial decline in the donkey population within China and the consequential need to import raw materials from other regions such as Africa, South America, and Australia (Maigari et al., 2020; Goodrum et al., 2022). Given the growing demand for ejiao and the diminishing supply of donkey hides, it becomes imperative to explore strategies to address this supply gap. To this end, the identification of candidate genes associated with collagen deposition in donkey skin emerges as a pivotal avenue of investigation. By cultivating new strains of Dezhou donkeys with enhanced skin performance through selective breeding, it may be possible to mitigate the scarcity of donkey hides and sustain the production of ejiao.

With the rapid development of molecular biology and related disciplines, animal breeding has moved from conventional breeding to molecular breeding. Marker-assisted selection and genomic selection have become mainstream practices in molecular breeding of livestock (Yang et al., 2017). Complex traits such as diseases, production parameters and skin development in animals are controlled by several genes. While RNA-seq is considered an emerging molecular technique utilizing for screening genes associated with complex traits (Wickramasinghe et al., 2014; Song et al., 2019). Consistently, by utilizing RNA-seq as a tool, several studies have been conducted in donkeys to screened key genes associated with skin thickness (Wang et al., 2022), skeletal muscles development (Li et al., 2022; Chai et al., 2023), and skin coat color (Wang et al., 2020).

So far very little information is available regarding the molecular mechanisms involve in the development of skin and collagen organization in Dezhou Donkeys. Thus, the current study endeavors to bridge this knowledge gap by employing RNA-Seq technology to analyze lncRNAs and mRNAs in the skin of Dezhou donkeys across different age groups. Furthermore, this study documented some key pathways like Protein digestion and absorption, PI3K-Akt signaling pathway, ECM-receptor interaction, and Relaxin signaling which have strong association with collagen restructuring and skin development in Dezhou donkey. Interestingly, our findings documented genes like *COL1A1* and *COL3A1* that were involved in the regulation of above mentioned pathways. Moreover, the role of LncRNAs (ENSEAST00005041187 and ENSEAST00005038497) showed a key role in regulation of *COL1A1* gene. Overall, our findings provided the foundational model for skin biology and collagen synthesis and organization in Dezhou donkeys’ skin.

## 2 Materials and methods

### 2.1 Ethical statement

The research conducted in this study adhered to stringent ethical guidelines and received the necessary approvals from the Animal Welfare and Ethics Committee of the Institute of Animal Sciences, Liaocheng University (Approval No. LC 2019-1). All aspects of the experimental procedures, including the use of experimental animals, were conducted in full compliance with local animal welfare laws, guidelines, and ethical codes. Our foremost commitment was to minimize any potential suffering experienced by the experimental animals throughout the study.

### 2.2 Experimental animals and sample collection

In this investigation, a cohort of nine male donkeys sourced from a reputable donkey farm located in Dezhou City, Shandong Province, was the subject of our study. To ensure a comprehensive analysis, the donkeys were stratified into three distinct age groups: 8-month-old fetuses, 2-year-olds, and 8-year-olds. Each age group was represented by three biological replicates. The 8-month-old fetuses were selected due to miscarriages resulting from external pressure, and skin samples were promptly collected within 1 h following miscarriage. In contrast, minimally invasive skin sampling techniques were employed for the 2-year-old and 8-year-old donkeys. All sampling locations were carefully chosen at the midpoint of the left dorsal region, situated between the sixth and seventh thoracic vertebrae. Before sampling, rigorous preparation of the sampling sites was conducted using a specialized skin preparatory instrument. Furthermore, to alleviate any potential pain, procaine was administered at the designated sampling points. Subsequently, a 5 mm skin sampler (Acuderm, United States) was employed to procure skin tissue samples, which were meticulously washed with phosphate-buffered saline (PBS) and then stored in cryotubes before rapid freezing in liquid nitrogen. These tissue samples were subsequently preserved at −80°C, awaiting RNA-Seq analysis. Following the sampling process, sterile gauze was applied to staunch any bleeding, and anti-inflammatory drugs, in conjunction with iodine, were administered for treatment purposes. It is essential to emphasize that all the donkeys included in this study were in a healthy condition and exhibited favorable prognoses.

### 2.3 RNA extraction and sequencing

To initiate the molecular analysis, the skin tissue samples were initially ground to a fine powder in liquid nitrogen. Total RNA extraction was then carried out using the Trizol reagent kit (Invitrogen, Carlsbad, CA, United States) as per the manufacturer’s guidelines. The quality of extracted RNA was assessed using the Agilent 2,100 Bioanalyzer (Agilent Technologies, Palo Alto, CA, United States), with RNase-free agarose gel electrophoresis employed for additional quality evaluation. Subsequent to total RNA extraction, eukaryotic mRNA was selectively enriched through the use of Oligo (dT) beads. The construction of cDNA libraries was accomplished by Gene *Denovo* Biotechnology Co. (Guangzhou, China), and Illumina Novaseq6000 was the chosen platform for sequencing.

### 2.4 Sequencing data quality control and read mapping

**FIGURE 1:**
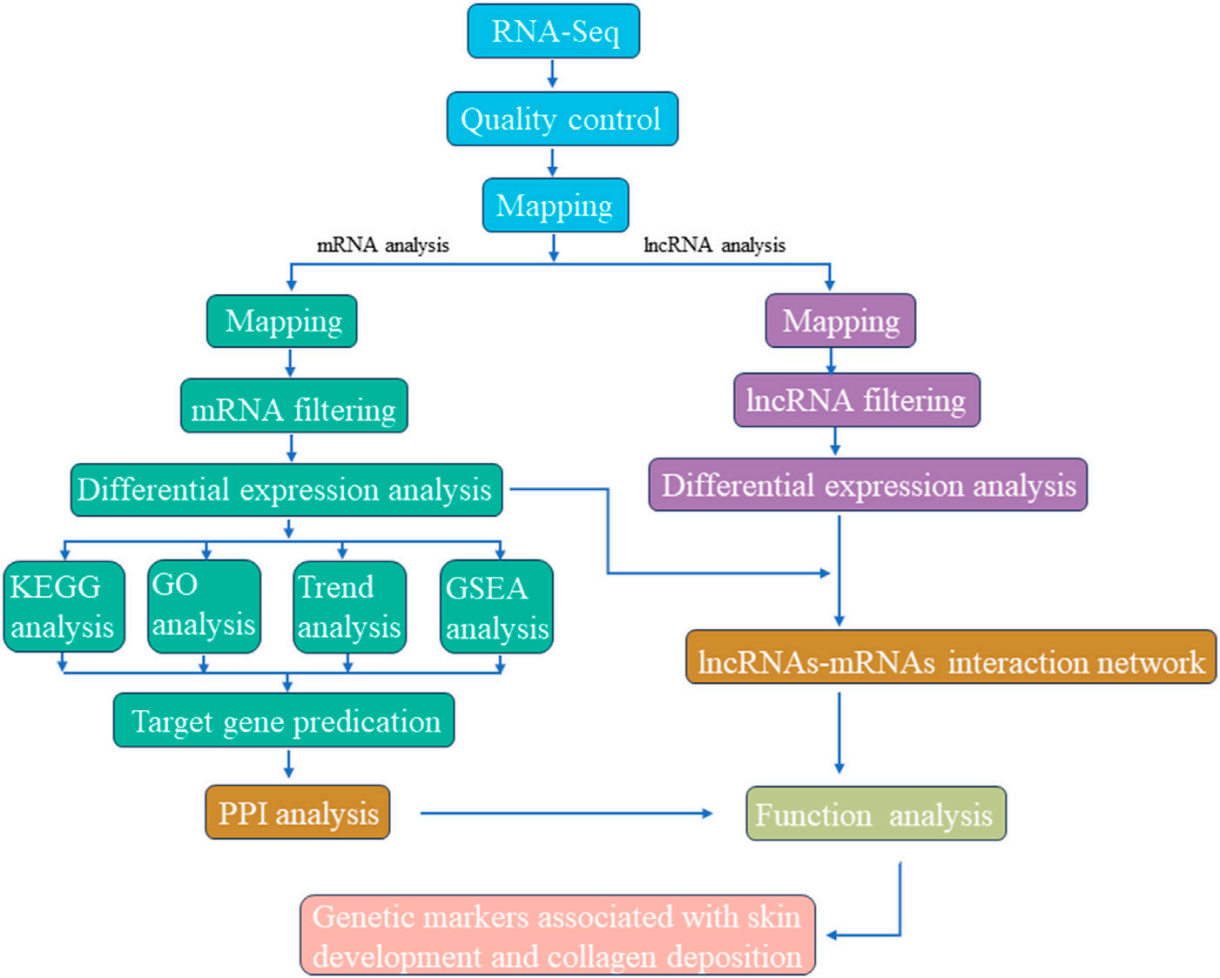
Graphical presentation of methodology adopted for screening candidate genetic markers associated with skin development and collagen deposition.

The overall RNA-seq protocol adopted for this study has been summarized in Figure 1. To ensure the reliability of our data, a series of rigorous quality control measures were instituted. Initial filtering of raw reads was accomplished using fastp (Chen et al., 2018) (version 0.18.0). Reads containing adapters, those with more than 10% unknown nucleotides (N), and those characterized by low quality, defined as having more than 50% bases with a quality score (q-value) of ≤20, were systematically removed from the dataset. Furthermore, Bowtie2 (Langmead and Salzberg, 2012) (version 2.2.8) was deployed to eliminate reads marked as rRNA, thereby resulting in a collection of high-quality clean reads ready for subsequent assembly and analysis. HISAT2.2.4 (Kim et al., 2015) was subsequently employed to align the paired-end clean reads with the reference genome of the Dezhou donkey (ASM1607732v2). The assembled reads from each sample were consolidated using StringTie v1.3.1 (Pertea et al., 2015; Pertea et al., 2016). The calculation of FPKM (fragment per kilobase of transcript per million mapped reads) values to quantify gene expression levels was facilitated by RSEM (Li and Dewey, 2011). Correlation analysis was executed using R, while principal component analysis (PCA) was carried out utilizing the gmodels package (http://www.rproject.org/). Differential expression analysis was undertaken using DESeq2 (Love et al., 2014) software, which was employed to identify differentially expressed genes (DEGs) meeting the criteria of a fold change ≥2.00 and an adjusted *p*-value of 0.05.

### 2.5 Identification and prediction of differentially expressed LncRNAs

Identification of potential lncRNAs was accomplished through a comprehensive multi-step process. Firstly, Gffcompare was employed to retain transcripts classified as class “u,” signifying intergenic transcripts (Shi et al., 2019a). Subsequently, based on the merged GTF file, only transcripts exhibiting characteristics such as more than one exon and a length exceeding 200bp were retained (Chen et al., 2019a; Mishra and Wang, 2021). Coding potential assessment of non-coding transcripts was executed using CPC2, CNCI, LGC, and PLEK, with the intersection of predictions being designated as novel lncRNAs (Chen et al., 2019; Mishra and Wang, 2021). To further enhance specificity, transcripts translated in all six possible frames were subjected to scrutiny using HMMER. Any transcripts displaying homology with known protein family domains in the Pfam database were excluded. Furthermore, the BLASTX program was harnessed to filter out transcripts bearing similarity to known proteins present in the NCBI nr and UniRef90 databases (E Value < 1e-5). Finally, transcripts with FPKM values exceeding zero in at least one sample were retained.

### 2.6 Potential target gene prediction and network construction

The identification of potential target genes was facilitated through the utilization of BEDTools (version 2.17.0) (Quinlan and Hall, 2010). Adjacent protein-coding genes situated within a 100 kb radius of each lncRNA locus were selected. Additionally, protein-coding genes exhibiting a Pearson correlation coefficient exceeding 0.95 with differentially expressed lncRNAs were considered as potential target genes. These target genes were subsequently integrated with the DEGs dataset. For a more comprehensive understanding of the interactions between DEGs and various groups, a protein-protein interaction (PPI) network was constructed employing the STRING database (https://string-db.org/) (Szklarczyk et al., 2015). Furthermore, an lncRNA-mRNA network was formulated based on targeting relationships, with visualization achieved through the Cytoscape software (V3.9.0) (The Cytoscape Consortium, United States), employing default parameters and the “layout = attribute circle layout” setting (Saito et al., 2012).

### 2.7 Functional enrichment analysis of DEGs and DELs

Functional enrichment analysis was conducted to gain insights into the biological relevance of DEGs and DELs. The GO database (Ashburner et al., 2000) was employed to predict molecular functions, cellular components, and biological processes associated with DEGs and DELs. Comparison with the Gene Ontology database (http://www.geneontology.org/) enabled mapping of all DEGs and DELs to their respective GO terms. Additionally, KEGG annotation (http://www.genome.jp/kegg) (Kanehisa and Goto, 2000) was used to subject DEGs and DELs to KEGG enrichment analysis. False discovery rate (FDR) correction, specifically employing the Benjamini–Hochberg adjustment method, was applied. GO and KEGG terms boasting *p* values below 0.05 were identified as significantly enriched.

## 3 Results

### 3.1 Overview of sequencing data in Dezhou donkey skin

**FIGURE 2:**
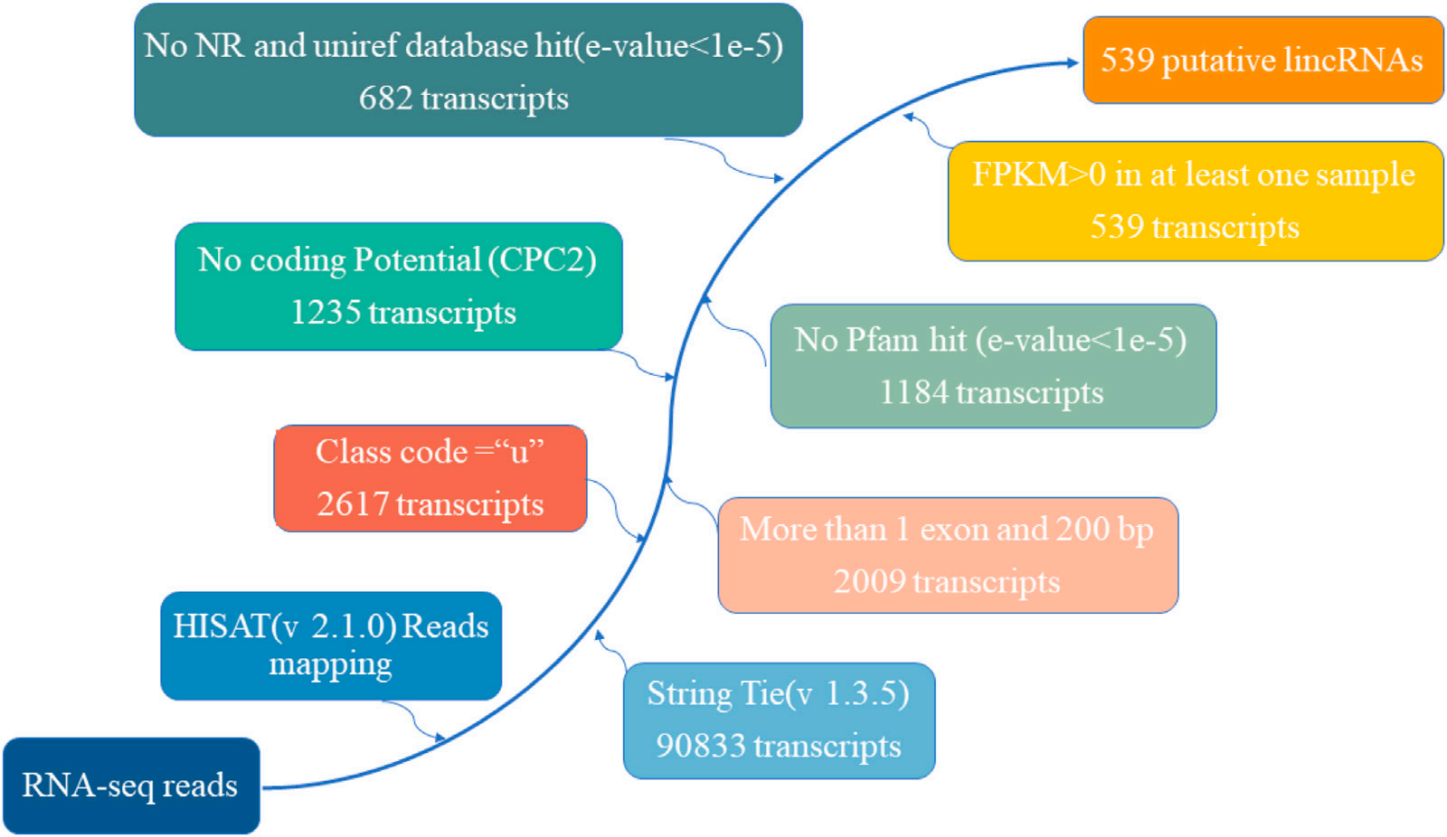
The pipeline for the identification of putative lncRNAs in this study, while the frames in the direction of the arrow show the filtering process and the number of screened transcripts.

A total of nine cDNA libraries were constructed, each representing a distinct time point or developmental stage. The raw data obtained from these libraries yielded an average of approximately 46,860,812 reads per sample. These raw reads were subjected to rigorous quality control measures, resulting in an average of 46,637,055 clean reads per sample. Notably, the clean reads constituted approximately 99.37% of the raw data, underscoring the high quality of the sequencing data. Furthermore, the quality assessment revealed that the clean reads possessed exceptional accuracy, with Q20 and Q30 percentages exceeding 97.15% and 92.26%, respectively (Supplementary Table S1). These metrics are indicative of the reliability and precision of the sequencing process. Subsequently, a critical step in the analysis involved mapping the clean reads to the reference genome of the Dezhou donkey (ASM1607732v2). Impressively, over 92.86% of the clean reads were successfully mapped to the reference genome. Among these mapped reads, approximately 76.11% aligned to the exon regions, approximately 12.75% to the intron regions, and approximately 11.14% to the intergenic regions. These mapping results provided essential information regarding the distribution of sequenced reads across different genomic regions (Table 1). To further elucidate the transcriptome profile, we employed StringTie to assemble transcripts for each library. Subsequently, all assembled transcripts were synthesized into a nonredundant transcript dataset using StringTie-Merge. This comprehensive transcriptome dataset served as a foundation for downstream analyses. Additionally, the study identified a subset of 539 putative long non-coding RNAs (lncRNAs) based on the criteria illustrated in Figure 2.

**TABLE 1:** Quality assessment of sequencing data.

Sample	RawDatas	CleanData (%)	Q20 (%)	Q30 (%)	Total_Mapped (%)	exon (%)	intron (%)	intergenic (%)
YD-1	45633838	99.46	97.59	93.20	96.34	74.81	14.34	10.85
YD-2	44955666	99.37	97.15	92.26	95.72	74.22	14.71	11.07
YD-3	48310130	99.48	97.49	92.98	96.17	75.90	13.48	10.62
MD-1	45978156	99.57	97.76	93.61	93.15	79.82	9.94	10.24
MD-2	50951222	99.56	97.66	93.37	92.85	76.79	12.10	11.11
MD-3	47430242	99.57	97.87	93.86	93.20	77.35	11.55	11.10
OD-1	43964952	99.62	97.42	92.75	92.63	75.48	13.24	11.28
OD-2	46192542	99.50	97.66	93.31	92.83	76.06	12.23	11.71
OD-3	48330560	99.56	97.73	93.45	92.97	74.59	13.16	12.24

### 3.2 Identification of differentially expressed mRNAs (DEMs) and long non-coding RNAs (DELs) in the Dezhou donkey

**FIGURE 3:**
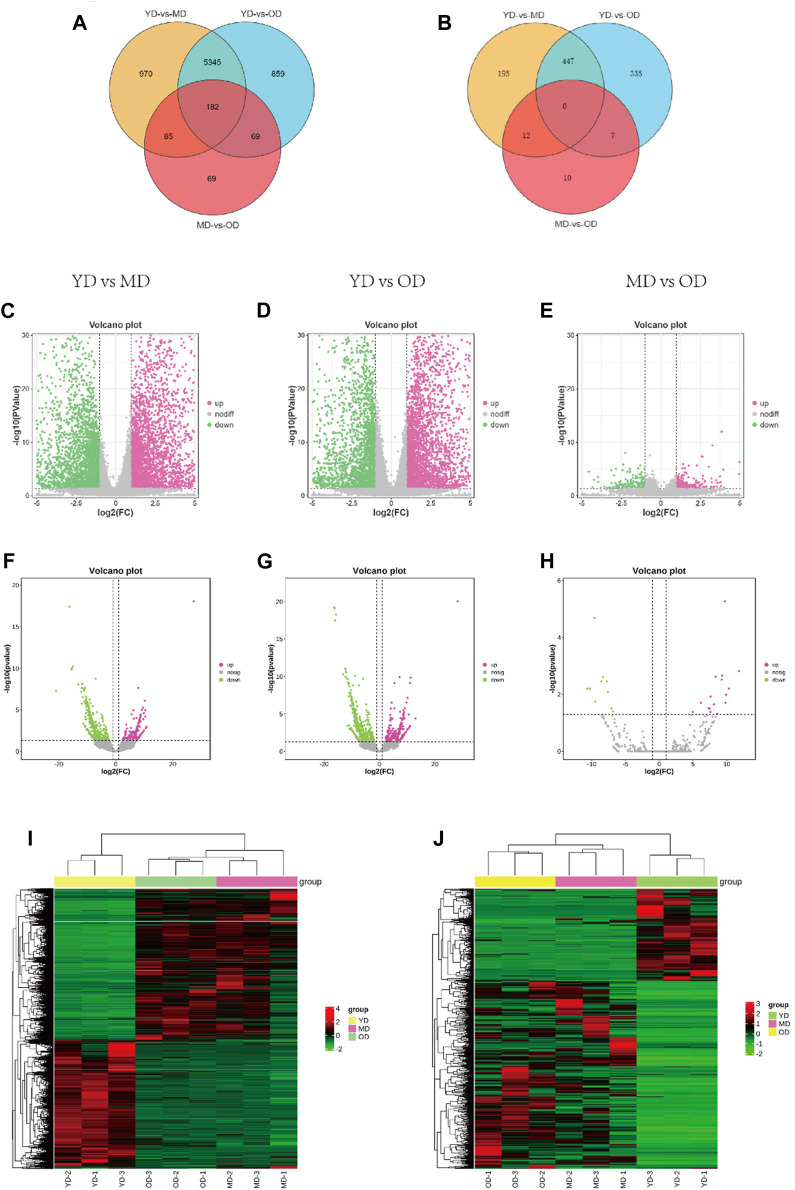
Analysis of DEMs and DELs expression profiles in the Dezhou donkey. **(A, B)** are the Venn diagrams of differentially expressed mRNAs and lncRNAs, respectively. **(C–H)** are the volcano plots of mRNAs and lncRNAs, respectively. **(I, J)** are the heatmaps of differentially expressed mRNAs and lncRNAs, respectively.

The overall data has been provided in Supplementary Table S2. In this section, we delve into the analysis of differentially expressed mRNAs (DEMs) and long non-coding RNAs (DELs) in the skin tissues of Dezhou donkeys across various comparative groups. Our approach involved stringent criteria for identifying these differential expressions based on fold change and statistical significance. Based on the criteria of |log_2_FC(fold change)| > 1 and q-value <0.05 normalized expression, total of 6,582, 6,455, and 405 differentially expressed mRNAs (DEMs) (Figures 3C–E) respectively. In addition, 654, 789, and 29 differentially expressed lncRNAs (DELs) were presented in the skin tissues of Dezhou donkeys among YD vs. MD, YD vs. OD, and MD vs. OD, respectively (Figures 3F–H). Furthermore, Venn diagram analysis reveals 182 differential genes were commonly shared among the three groups (YD vs. MD, YD vs. OD, and MD vs. OD) (Figures 3A, B).

To provide a comprehensive visualization of the differential expression patterns in both mRNAs and lncRNAs, clustered heatmaps were constructed. These heatmaps offer a clear representation of the distinct expression profiles among the identified DEMs and DELs. The clustering methodology utilized here groups genes with comparable expression patterns, as visually depicted in Figure 3I (mRNAs) and Figure 3J (lncRNAs).

### 3.3 GO and KEGG enrichment analysis of DEGs

**FIGURE 4:**
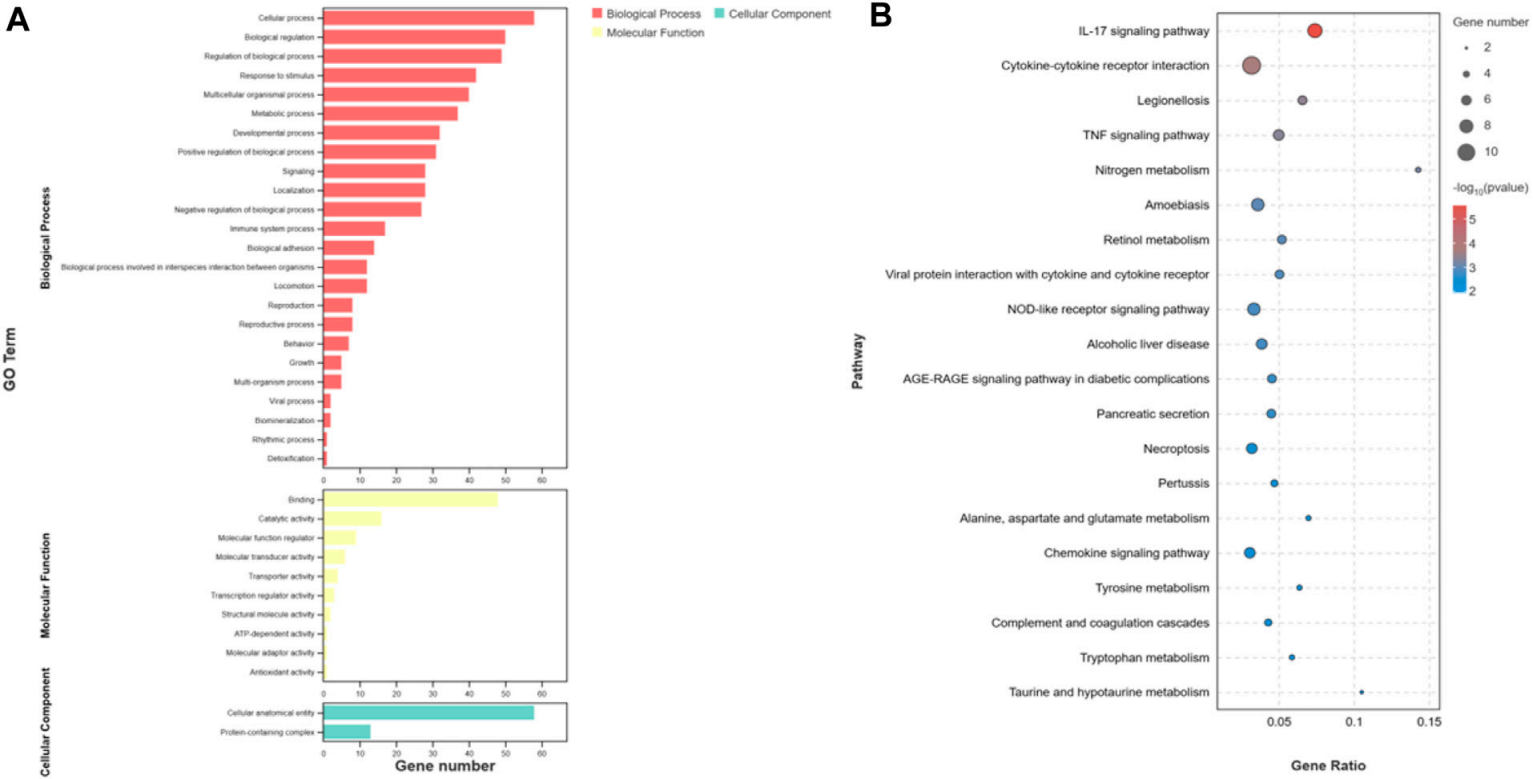
Functional analysis of differentially expressed mRNAs. **(A)** is the bar graph showing the enrichment of GO terms and the number of genes involved in differentially expressed mRNAs. **(B)** represents the top 20 enriched KEGG pathways for differentially expressed mRNAs.

The overall data obtained for pathways and functional processes [(Biological Processes (BP), Molecular Functions (MF), and Cellular Components (CC)] has been presented in Supplementary Table S3. To investigate the functions and pathways of DEGs, we conducted separate analyses using GO terms and KEGG pathways. According to the analysis of GO terms for the DEMs, we identified a total of 182 DEMs that were significantly enriched in 36 GO terms. These terms were further categorized into 24 Biological Processes (BP), 10 Molecular Functions (MF), and 2 Cellular Components (CC). Notably, the Biological Processes were primarily associated with cellular processes, metabolic processes, and biological regulation. In the Molecular Functions category, the top terms included binding, catalytic activity, and molecular function regulation. Our GO analysis of Cellular Components indicated that the DEMs were enriched in two specific GO terms, namely, cellular anatomical entity and protein-containing complex (Figure 4A). Furthermore, the KEGG pathway analysis unveiled a total of 168 pathways. Among these, 45 pathways demonstrated significant enrichment (*p*-value <0.05) (Figure 4B). In addition, based on bioinformatics analysis, we selected the pathways and biological function processes associated with protein metabolism and skin development.

**FIGURE 5:**
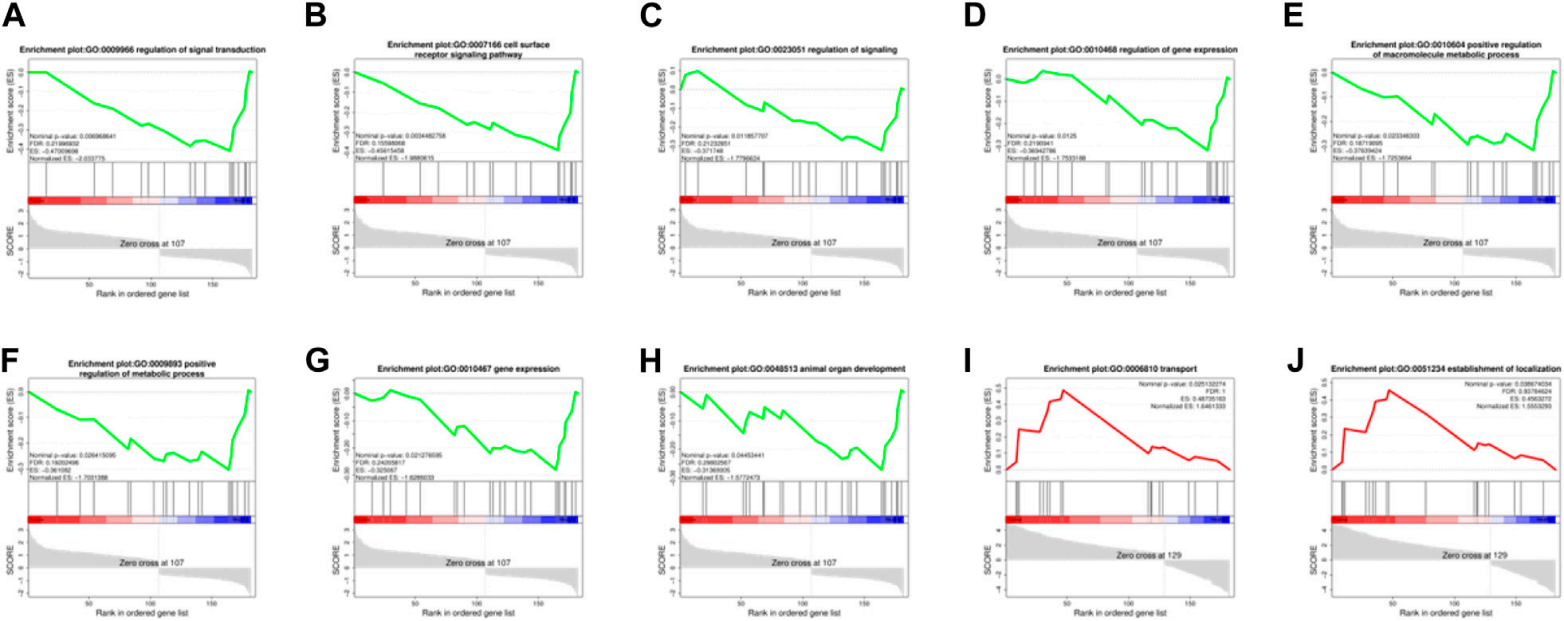
Enrichment Analysis (GSEA) analysis on three groups of 182 differentially expressed genes. **(A–H)**: The significantly enriched GO terms related to protein synthesis during the MD vs. OD period (nominal *p*-value<0.05). **(I, J)**: The significantly enriched GO terms related to protein synthesis during the YD vs. OD period (nominal *p*-value<0.05).

**FIGURE 6:**
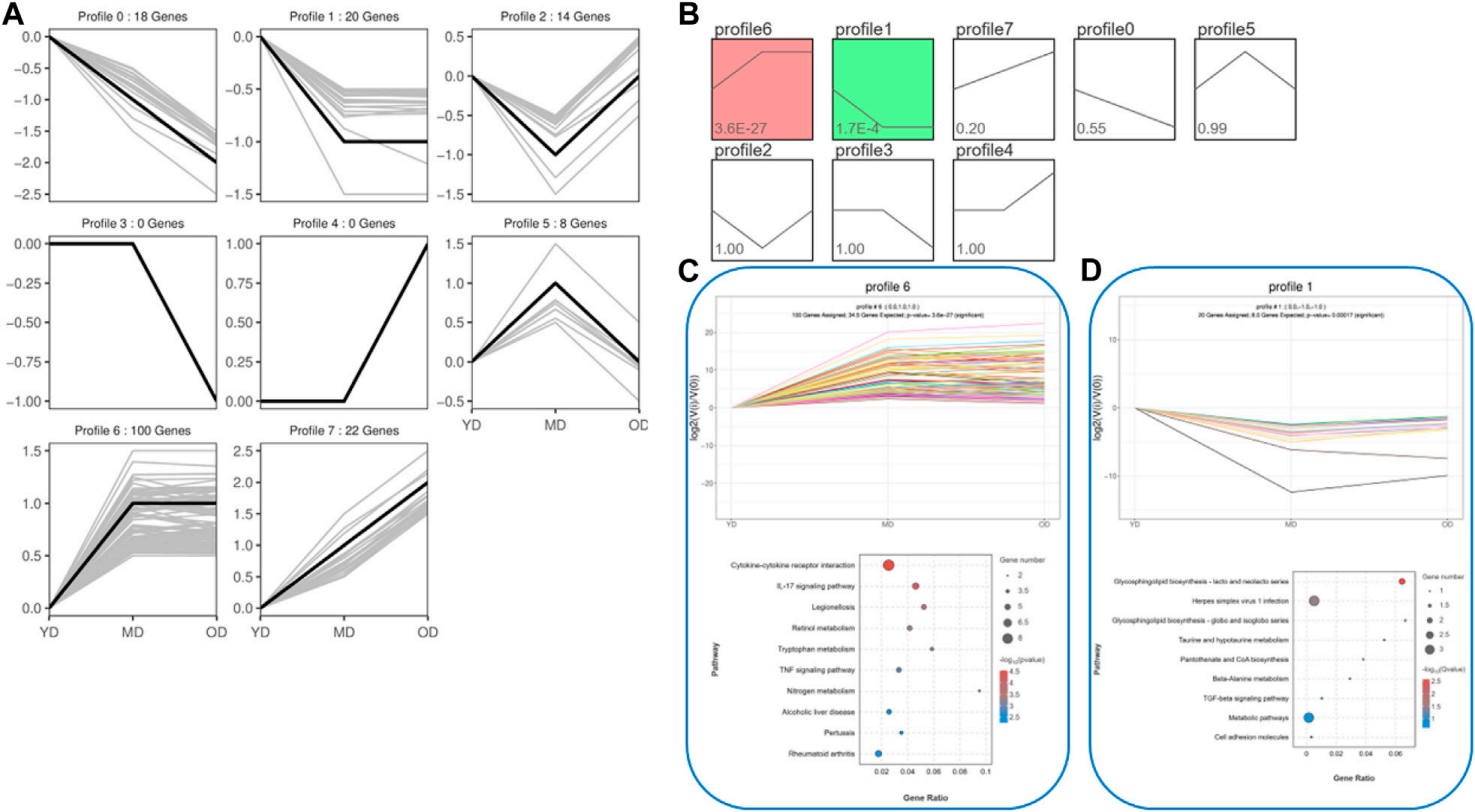
The trend analysis was performed on the target gene group. **(A)** Actual trend of gene mutations. **(B)** Trend graph fitted based on the predetermined number of trends and the genetic change trend. **(C)** KEGG functional analysis of profile 6 genes reveals the top 10 significantly enriched pathways. **(D)** KEGG functional analysis of profile 1 genes reveals the top 10 significantly enriched pathways. By conducting the aforementioned analysis on the DEMs, several candidate genes (*ELAPOR1, FOSL1, MEP1B, PAX9, PPP1R1B, SIX4, ZEP36, CD14, COL1A1, COL3A1, COL6A5, EGFL6, PGLYRP4, SERPINB13,* and *Spink6*) associated with protein synthesis in the skin were identified.

In order to effectively address the limitations of traditional enrichment analysis in mining relevant information for low-effect genes, we conducted Gene Set Enrichment Analysis (GSEA) analysis on three groups of differentially expressed genes that were co-expressed (Figure 5). Through this analysis, we identified significant enrichment of GO terms related to protein synthesis (Supplementary Table S4). At the same time, a trend analysis was performed on the target gene group (Figure 6; Supplementary Table S5), which was classified into six profiles (excluding profile 3 and 4). Enrichment analysis was conducted on these profiles, and the results indicated a significant association between the expression of numerous genes and pathways such as Cell growth and death, Signal transduction, metabolic process, immune system, and signal molecules and interaction.

### 3.4 PPI analysis of the DEMs related to protein deposition in the Dezhou donkey skin

**FIGURE 7:**
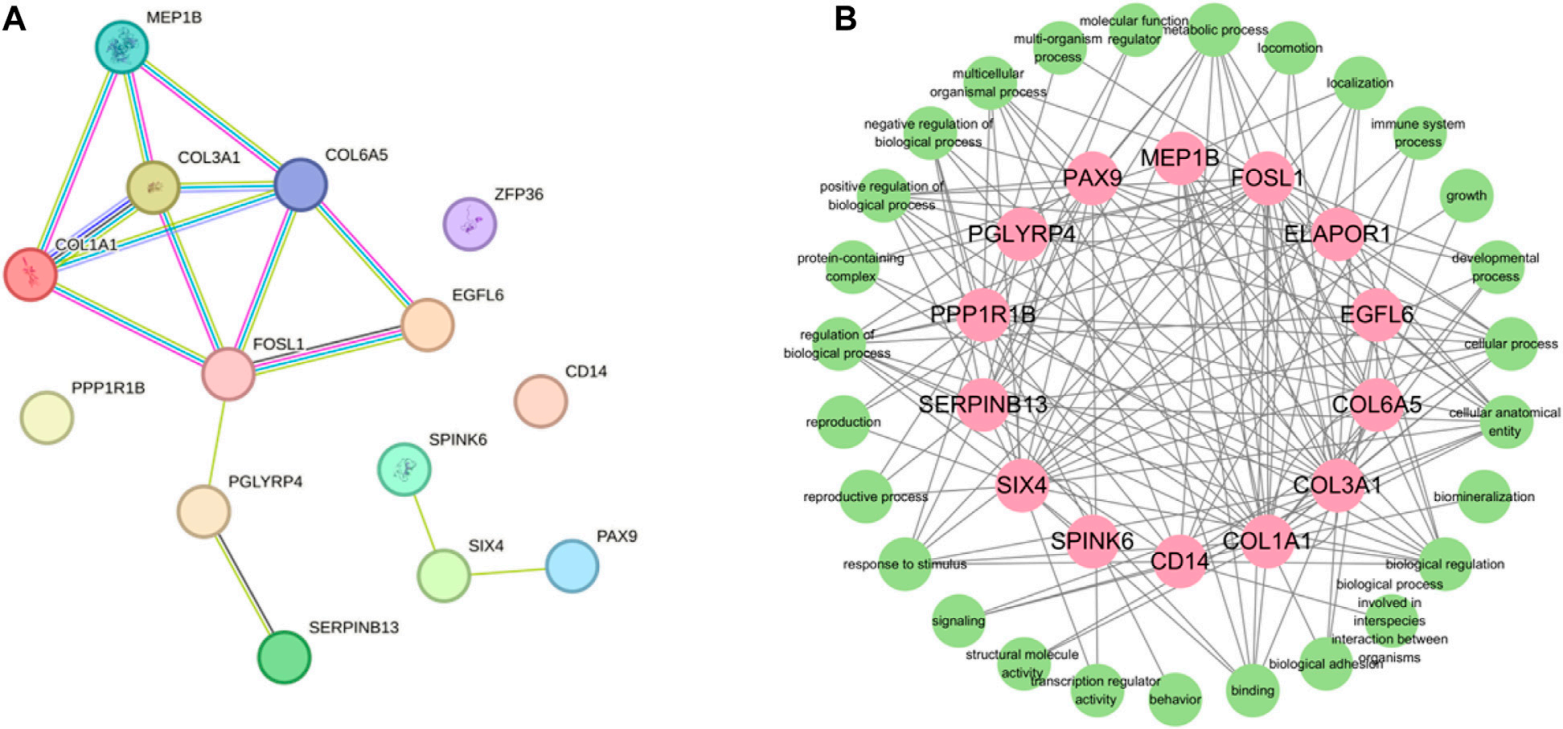
Protein-protein interactin (PPI)network. **(A)** PPI network was constructed by the STRING database using the protein synthesis candidate genes. **(B)** GO terms association map of protein synthesis candidate genes.

In order to gain comprehensive insights of the biological processes associated with protein synthesis candidate genes, we concentrated on the posttranslational protein levels of the candidate genes. Therefore, we constructed protein-protein interaction networks (PPIs) by the STRING (https://string-db.org/STRING, accessed on 10 April 2022). There were 14 nodes and 15 edges in the PPI network (Figure 7). According to the results of the PPI network analysis, *COL3A1* was the hup gene (degree = 14). In addition, PPI network analysis of candidate genes was significantly enriched in GO terms, including fibrillar collagen trimer and collagen-containing extracellular matrix. *FOSL1, PGLYRP4,* and *SIX4* were the key degrees of the PPI network with other proteins.

### 3.5 Analysis of the targeting relationship between lncRNAs and mRNAs

**FIGURE 8:**
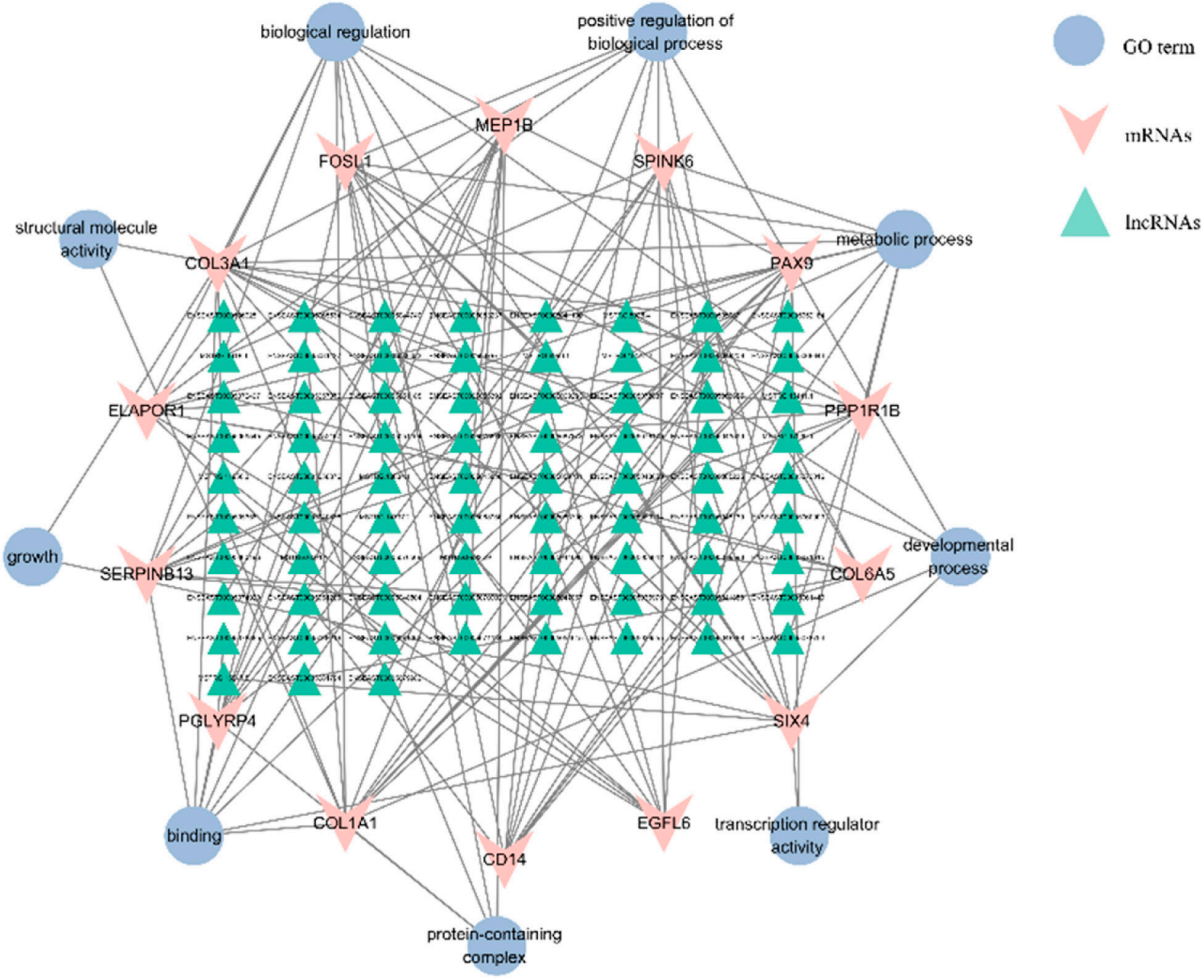
The interaction networks of “pathways-differentially expressed lncRNAs-target genes”. The triangle is for lncRNA, the “V” represents the target gene, and the ellipse is for the GO terms of the target gene, respectively. A total of 93 DELs were regulated with 14 mRNAs in the interactive network.

The DEMs and DELs with Pearson correlation coefficients above 0.95 were selected to construct the lncRNA-mRNA co-expression network using Cytoscape v.3.10.1. In this network, a total of 739 nodes and 2,428 lncRNA-mRNA pairs were identified based on the results (Supplementary Table S6). There were 75 lncRNAs corresponding to 14 target gene in Figure 8. Enrichment analysis using GO terms was conducted to understand the functional implications of these lncRNAs and their associated target genes. From Figure 8, it could be observed that the target genes were mainly enriched in protein synthesis and metabolic, such as binding, protein-containing complex, biological regulation, and metabolic process. This indicated that lncRNAs could interfere with target genes, thereby regulating protein synthesis function in donkey skin. LncRNAs played a crucial role in regulating the functions of some genes in different pathways. We observed that 15 target genes (*ELAPOR1, FOSL1, MEP1B, PAX9, PPP1R1B, SIX4, ZEP36, CD14, COL1A1, COL3A1, COL6A5, EGFL6, PGLYRP4, SERPINB13,* and *Spink6*.) were regulated by lncRNAs to function in protein synthesis and metabolic, although these DEMs participated in other pathways. The collagen proteins encoded by *COL1A1* and *COL3A1* were involved in the synthesis and repair processes of the extracellular matrix in the extracellular matrix remodeling pathway. Enrichment analysis of lncRNA-related mRNAs was performed through GO terms to understand the functions of these lncRNAs and target genes. It can be seen from Figure 8, the target genes are mainly enriched in protein synthesis and metabolic. This indicates that lncRNA can interfere with target genes, thereby regulating the protein synthesis function in donkey skin.

## 4 Discussion

In this study we have considered rigorous exploration of the transcriptome expression profiles, encompassing both mRNA and lncRNA, within Dezhou donkey skin, with a particular focus on collagen deposition at distinct developmental stages. The employment of RNA-seq technology has facilitated this comprehensive investigation. This rigorous inquiry has culminated in the identification of a total of 182 differentially expressed genes that were commonly altered across the three experimental groups. Some of these identified genes are postulated to wield substantial influence in governing the process of skin collagen deposition and development. Collagen, being a ubiquitous protein constituent across mammalian species, assumes a multifaceted role, being deposited within diverse organs and tissues, while simultaneously playing a pivotal role in both structural and functional aspects (Franchi et al., 2007; Devos et al., 2023). Consistently, it has been well-established that the process of collagen deposition is orchestrated through a complex interplay of gene regulatory networks (Reilly and Lozano, 2021).

In this study, we also observed that the 182 DEMs exhibit significant enrichment across 168 distinct pathways. Notably, several of these pathways hold direct relevance to the intricate process of skin collagen deposition and development. Of these, Protein digestion and absorption, Metabolic pathways, PI3K-Akt signaling pathway, ECM-receptor interaction, and Relaxin signaling showed their pivotal roles in orchestrating collagen deposition within the dermal matrix, skin development, extracellular matrix and collagen fibril organization of Dezhou donkey skin (Table 2). Interestingly, a recent study found several important signaling pathways related to wool growth and bending, such as ECM-receptor interaction, PI3K-Akt signaling pathway, Relaxin signaling pathway, protein digestion and absorption, and metabolic pathways in Zhongwei goats (He et al., 2020; Liu et al., 2022). In addition, a recent experimental trial has shown that PI3K-Akt signaling pathway, Protein digestion and absorption and ECM−receptor interaction signaling pathways were associated with regulation of actin filament-based process, regulation of actin cytoskeleton organization, cell-matrix adhesion and collagen fibril organization (Wang and Gu, 2021). Furthermore, they assumed that the aging of skin might be associated with extracellular matrices depletion, which plays a key role in many cellular processes including migration, differentiation, and survival (Bonnans et al., 2014). Combined with published article data and our current findings it was proved that these pathways may exert vital roles in skin development, collagen and extracellular matrix organization.

**TABLE 2:** Selected Pathways/Biological processes associated with metabolism, collagen organization and skin development.

Pathways	Genes count	*p*-value	Genes name
Protein digestion and absorption	4	1.72E-04	COL1A1, COL3A1, MEP1B, COL6A5
Relaxin signaling pathway	3	0.008505048	COL1A1, COL3A1, RXFP1
Metabolic pathways	6	0.018227888	B3GALT2, CTH, FMO2, FUT2, LOC106840130, GLUL
Glycosphingolipid biosynthesis - lacto and neolacto series	2	0.032318823	B3GALT2, FUT2
Alanine, aspartate and glutamate metabolism	2	0.034689964	LOC106840130, GLUL
Cysteine and methionine metabolism	2	0.038261718	CTH, LOC106840130
Biosynthesis of amino acids	2	0.048155606	CTH, GLUL
ECM-receptor interaction	2	0.043557123	COL1A1, COL6A5
Biological Processes			
skin development	2	0.005881289	COL1A1, COL3A1
collagen fibril organization	2	0.007658935	COL1A1, COL3A1
cellular response to amino acid stimulus	2	0.007658935	COL1A1, COL3A1
extracellular matrix organization	2	0.025318973	COL1A1, COL3A1

**FIGURE 9:**
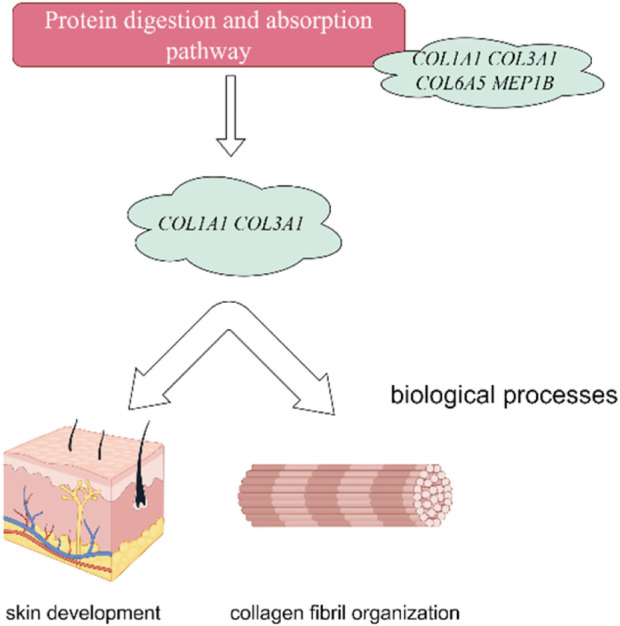
Collagen Alpha family genes regulate protein digestion and absorption signaling pathway. Furthermore, the protein digestion and absorption signaling pathway showed their association with skin development and collagen organization in Dezhou skin. The figure has generated via Figdraw.

In this investigation, we have delved into the intricate landscape of collagen synthesis and its associated genetic regulators. Specifically, *COL1A1*, *COL3A1*, *COL6A5* and *LOC106840130* have been identified as pivotal players in regulation of important pathways including ECM-receptor interaction, Relaxin signaling pathway, and protein digestion and absorption and biological function processes (skin development, extracellular matrix and collagen fibril organization) as shown in Figure 9. Collagen, a prominent member of the extracellular matrix (ECM), is characterized by its triple-helix structure (Devos et al., 2023). Extensive research has showcased the widespread presence of collagen I, collagen II, and collagen III across various anatomical domains in numerous animal species, encompassing skin, skeletal structures, blood vessels, tendons, and internal organs (Rahkonen et al., 2004; Ben Amor et al., 2013; Wang et al., 2017). Notably, prior investigations have unveiled the profound impact of collagen peptide and vitamin C derivative co-treatment on upregulating *COL1A1* and *Has2* expression, thereby averting skin thinning (Shibuya et al., 2014). Furthermore, single nucleotide polymorphisms within the *COL3A1* and *COL6A5* genes have been associated with atopic dermatitis, revealing distinct genotype-phenotype relationships (Szalus et al., 2023). These genetic variations within these collagen-related genes may exert substantial influence on collagen expression, structure, and function, thereby modulating the pathogenesis and clinical manifestations of atopic dermatitis (Söderhäll et al., 2007). Consistently, it has been revealed that collagen alpha family genes play a key role in skin aging in human (Wang and Gu, 2021), and hair follicular stem cell development in goat (Reilly and Lozano, 2021) by regulating extracellular matrix and collagen organization respectively.

Concurrently, our study has unveiled the role of the *LOXL2* gene among the differentially expressed genes, shedding light on its involvement in collagen synthesis and modification. The multifaceted functions of *LOXL2* encompass gene transcription, cell motility, migration, adhesion, angiogenesis, and differentiation (Fujimoto and Tajima, 2009). Intriguingly, the present investigation has observed a significant downregulation of *COL1A1*, *COL3A1*, and *LOXL2* genes with increasing age, signifying the potential involvement of myriad molecular mechanisms, including epigenetic regulation, alterations in intracellular signaling pathways, and fluctuations in hormone levels (Wang et al., 2021; Wang and Gu, 2021). However, the precise mechanistic underpinnings necessitate further comprehensive research to validate and elucidate.

It is imperative to acknowledge that previous studies have elucidated the regulatory influence of lncRNAs in a cis manner, directly impacting gene expression (Zou et al., 2017; Shi et al., 2019b; Chen et al., 2019c; Zhu et al., 2020). In this study, DEL ENSEAST00005067788 was observed to exert an influence on *COL1A1* and *COL3A1* genes, with significantly higher expression levels in the YD period, consistent with the observed expression trends. This implies that lncRNA may modulate target gene expression by interfering with regulatory regions on the same chromosome. Particularly noteworthy is the involvement of other DELs, including ENSEAST00005041187, ENSEAST00005038497, MSTRG.17248.1, and other lncRNAs, in the cis-regulation of COL1A1 gene expression, emphasizing their potential roles in protein binding or the regulation of protein-containing complexes (Liu et al., 2016). Furthermore, the *LOXL2* gene, highly expressed in the YD period, was similarly cis-regulated by lncRNAs such as MSTRG.7943.1 and ENSEAST00005067788. Mutations within the *LOXL2* gene locus have been linked to decreased elastin renewal, underscoring its pivotal role in skin physiology (Gambichler et al., 2019). Moreover, hypermethylation of the *LOXL2* gene promoter region has been associated with elastolytic conditions, further highlighting its significance (Gambichler et al., 2016).

In summary, this research has elucidated the regulatory interplay between DELs and DEMs in the context of skin biology, with potential implications for collagen deposition in donkey skin. The identified genetic and epigenetic factors, including *COL1A1*, *COL3A1*, *LOXL2*, and various lncRNAs, may serve as key orchestrators in modulating the intricate processes underpinning collagen synthesis and deposition. This comprehensive investigation not only enhances our understanding of the molecular mechanisms governing collagen homeostasis but also paves the way for further research into therapeutic interventions and clinical applications in the field of dermatology and tissue engineering. Although our study provides important insights into the candidate genes related to collagen deposition in the skin of Dezhou donkeys, our study still has some limitations. First, our study mainly focuses on the analysis at the gene level, and we have not been able to deeply explore how these genes affect the deposition of collagen at the protein level. Secondly, our study was only conducted in Dezhou donkeys and did not consider other breeds of donkeys. For future research, we suggest further studying the role of these candidate genes at the protein level and their role in other donkey breeds.

## 5 Conclusion

In conclusion, the investigation into the transcriptome of Dezhou donkey skin during the Young Donkey (YD), Middle Donkey (MD), and Old Donkey (OD) periods has unveiled notable variations in the regulation of both mRNAs and lncRNAs specifically associated with collagen deposition. Our analysis, guided by GO annotations and KEGG, has highlighted a substantial proportion of DEGs such as coolagen alpha family genes *COL1A1*, *COL3A1*) and *LOXL2* are intricately linked to important signaling pathways (Protein digestion and absorption, PI3K-Akt signaling pathway, ECM-receptor interaction, and Relaxin pathways) and biological function processes (skin development, extracellular matrix and collagen fibril organization). In addition, Furthermore, the construction of an interaction network encompassing lncRNA genes has illuminated the potential roles of certain lncRNAs (ENSEAST00005041187, ENSEAST00005038497, MSTRG.17248.1) in modulating target genes (*COL1A*), thereby contributing to the intricate process of collagen deposition within the skin. The findings presented herein not only expand our comprehension of the regulatory networks governing collagen organization and skin development but also furnish a foundational framework upon which further research endeavors in this domain can be grounded. This research, thus, plays a vital cornerstone for future explorations aimed at advancing our knowledge of skin biology and collagen synthesis and organization in Dezhou donkeys.

## Data Availability

The data presented in the study are deposited in the SRA repository, accession number PRJNA1069658.
